# How Do Disadvantaged Children Perceive, Understand and Experience Household Food Insecurity?

**DOI:** 10.3390/ijerph18084039

**Published:** 2021-04-12

**Authors:** Stefania Velardo, Christina M. Pollard, Jessica Shipman, Sue Booth

**Affiliations:** 1 College of Education, Psychology & Social Work, Flinders University, GPO Box 2100, Adelaide 5001, Australia; 2 School of Public Health, Curtin University, GPO Box U1987, Perth 6845, Australia; C.Pollard@curtin.edu.au; 3 College of Nursing & Health Sciences, Flinders University, GPO Box 2100, Adelaide 5001, Australia; jessie.shipman@flinders.edu.au; 4 College of Medicine & Public Health, Flinders University, GPO Box 2100, Adelaide 5001, Australia; sue.booth@flinders.edu.au

**Keywords:** child-centred, children, disadvantage, food insecurity, qualitative

## Abstract

Food insecurity is associated with reduced physical, social, and psychological functioning in children. There has been sparse research into child food insecurity that incorporates children’s own perspectives, as adults are often interviewed as child proxies. While a nuanced, child-centred understanding of food insecurity is needed to inform effective policy and program responses, little is known about Australian children’s firsthand understanding or experience of household food insecurity. This study aimed to fill this gap by inviting preadolescent children’s perspectives. Eleven participants aged 10–13 years (seven girls and four boys) took part in the study and were recruited from an Australian charity school holiday camp that targets severely disadvantaged youth. Children took part in individual semi-structured interviews that incorporated drawings and emoji scales. Qualitative interviews were audio recorded, transcribed, and analysed using thematic techniques. Four themes emerged from the data analysis, children had: (i) financial understanding; (ii) awareness of food insecurity and coping mechanisms; (iii) sharing, empathy, and compassion for food insecure families; and (iv) described the nature of ‘food’ preparation. This study provides a child-centric analysis, demonstrating how children’s agency is enacted and constrained in food insecure contexts. This child-derived understanding of food insecurity provides a critical basis from which to build effective approaches to assess and respond to this significant social issue.

## 1. Introduction

Food insecurity refers to the “limited or uncertain availability of nutritionally adequate and safe foods or limited or uncertain ability to acquire acceptable foods in socially acceptable ways” [1]. Despite being a wealthy country, many Australians experience food insecurity, underpinned by rising levels of inequality [2]. Approximately 900,000 Australians live in a household that “in the previous 12 months had run out of food and been unable to buy more” in 2013, according to the latest national survey [3]. However, the single item measure used in the national surveillance survey is likely to underestimate the prevalence of food insecurity in Australia [4,5]. Poor or inconsistent surveillance of food insecurity in some developed countries has prompted urgent calls for more robust and routine systems [6].

Although Australia does not monitor the prevalence of food insecurity among children, available data suggests the number of Australian children vulnerable to food insecurity is high [7]. In 2017, the United Nations International Children’s Emergency Fund estimated that 16% of Australian children aged younger than 15 years lived with an adult who is moderately food insecure, and 4.9% lived with an adult who is severely food insecure [8]. A 2017 study of the prevalence of food insecurity among 9–16-year-old children residing in regional and remote Western Australia reported that about 20% were food insecure [9]. In 2019, a Western Australian survey of 100 socioeconomically disadvantaged families found that 80% had low or very low food security and 67% of adults said they were unable to feed their children a balanced meal because they could not afford to, 27% had cut the size of their children’s meals over the last year, and 13% said at least one of their children regularly skipped meals because there was not enough money for food in the household [10].

Food insecurity poses elevated risks to children’s health, wellbeing, and quality of life [11,12]. For children, developmental milestones can be hindered through poor nutrition due to poor diet quality and inadequate intake of essential nutrients [13]. Food insecure children have a higher propensity to consume cheap energy-dense food, resulting in increased dietary fat and sugar, and lower vegetable intakes than those who are food secure, which increases their risk of becoming overweight [14,15].

Food insecurity can also affect young people’s ability to engage with education. Specifically, school-age children residing in households experiencing food insecurity demonstrate compromised academic, behavioural, and psychosocial outcomes [12,16]. This includes higher rates of school absenteeism [17], increased likelihood of borderline or atypical emotional symptoms [16], and social and behavioural difficulties [16,18]. Hungry children with low nutritional intakes show poorer attendance, punctuality, and significantly lower grades at school [19].

For families, the level of ‘toxic’ or overwhelming stress associated with economic hardship can have long term emotional impact [20]. For example, Australian low to middle income families who were food insecure reported that it can trigger physical and psychological stress for parents due to them experiencing shame and concern about not being able to purchase food for their children [21]. Furthermore, Hecht et.al [22] argue that the experience of food insecurity should be recognised as a form of trauma, as an experience that is emotionally painful, often resulting on long term mental and physical consequences. The experience of food insecurity-related trauma is also likely to influence children. While shame and stigma are well described in the literature on adult food insecurity, less is known about children’s firsthand experiences of food insecurity. Research to date appears to have prioritised the assessment of adult populations as proxies for children, on the basis that adults are responsible for food provision and preparation, and that children may not understand the concepts associated with food insecurity. This approach is problematic as parents could inaccurately evaluate their children’s experiences of food insecurity [14,23]. An adult-centric perspective does not acknowledge that children may be active in their own struggle against chronic material deprivation and poverty. As Harvey asserts, children are best placed to describe their experiences of food insecurity [24].

Only a small number of studies to date, conducted in the United States, England, and Lebanon, have had children describe their lived experiences of food insecurity [14,23,24,25,26,27]. Collectively, these studies indicate that school-aged children aged seven-16 years can identify and discuss situations of food insecurity, by openly discussing their family’s limited ability to purchase certain foods due to high cost of living. Children across these studies described feelings of hunger, shame, and physical discomfort as the negative consequences of having less to eat. They also alluded to parental anxiety and shame in relation to household food insecurity, and reported their own feelings of worry, frustration, and empathy. The studies shed some light on coping strategies adopted by children to manage food insecurity, including decreasing food consumption, eating faster, and contributing to family finances.

Recent South Australian obesity intervention research unexpectedly highlighted children’s experiences of food insecurity [28]. In the 2017 study, children living in food insecure environments navigated the embodied and political aspects of feeling hungry. Indeed, Gunson et al. argue that the trauma and hunger resulting from food insecurity have social relevance in terms of how children experience relationships with others and how they are marked out in relation to social class [28]. These findings highlighted the need to engage in research with children that is mindful of the multi-faceted nature of food insecurity.

School holiday periods can be particularly stressful for low-income households with additional expenses and financial pressures incurred in providing care [29]. Children from disadvantaged families may also experience boredom, isolation, and heightened food insecurity during these periods, which can impact negatively on health and wellbeing [30]. To address this gap, a range of not-for-profit service providers aim to offer high-quality school holiday camps for disadvantaged children in countries like Australia [28] and the UK [30].

The collection of child-centred research is noteworthy in the degree to which it represents children’s unique experiences, distinct from those of the parents or other adults. There is an established body of childhood studies research that demonstrates the ways in which children enact agency in their day to day lives [31,32]. Yet, Punch argues that there is limited exploration of age or generation as a social variable in the same way that gender, race, and class have been theorised in relation to experiences of social issues [33]. Children’s food practices are ‘culturally and historically located’ and need to be understood in the context of ‘ongoing structuring and restructuring of the everyday social relations that take place between adults and children’ [34] (p. 7). It is this call for a critical theorization of childhood that we bring to our analysis in this paper.

Theoretically, this research drew inspiration from the New Social Studies of Childhood (NSSC) philosophy [35]. Historically, children have been excluded from research and overshadowed by adult accounts, despite their right to have their voices heard in matters that affect them [36]. This right is enshrined in the United Nations Conventions on the Rights of the Child which states that “young people have a right to participate in matters affecting them” [37]. The NSSC perspective challenges the traditional view of children as incompetent beings by privileging children’s voices and positioning them as capable of making a unique contribution to research [35].

This research aimed to describe children’s perceptions and experiences of food insecurity and its impact. In this paper, we explore the ways in which Australian pre-adolescent children living with financial hardship perceive and understand food insecurity and situate those experiences in the context of socially constructed understandings of child-adult relations. The research describes the perspectives of participants of a charity vacation camp for disadvantaged children conducted during the Australian school holidays in 2018.

## 2. Materials and Methods

### 2.1. Procedure

This study adopted a qualitative, interpretive approach to explore child food insecurity, by combining individual semi-structured interviews with visual tools (i.e., drawings and an emoji scale—please see Data collection section). Substantial insight can be gained by integrating visual and participatory methods when working with children, particularly when research is related to highly stigmatised or sensitive issues such as personal wellbeing, hunger and food insecurity [38,39]. In line with the work of Gunson et al., we did not incorporate visual methods to uncover a more ‘authentic truth’ of children’s experience [38]. Rather, drawings were used to assist in making qualitative interviewing less confronting for children, to increase children’s sense of agency in the research process, and as an additional lens through which to explore children’s perceptions [40]. Combining visual methods with interviews improves accessibility for young participants, rather than reinforcing deficit models about knowledge of food and eating [39]. The study was conducted in collaboration with Kickstart for Kids, an Australian charity that provides support to disadvantaged children through free school breakfast programs and vacation camps. Eligible study participants were children aged 10–13 years attending Camp Kickstart, a free one-week vacation program run in Adelaide. This age group was selected on the basis that children begin to assert more independence over food choices as they approach adolescence. Permission to recruit from the camps was obtained in writing from the Chief Executive Officer of Kickstart for Kids. Prior to recruitment, two researchers (SV and SB) attended the organization’s volunteer training program. They then contacted camp coordinators from two metropolitan sites and sought permission to recruit participants. Children were invited to participate in the study over three school holiday periods. The researchers spent time at each camp participating in unrelated activities with the children to develop trust and rapport and ground the study in the context of the participants [41].

Close attention was paid to the issue of consent and ensuring children and their parents understood what the research entailed. During recruitment, children were presented with a child-friendly information pamphlet, with a tear-off consent form. In small group presentations, the researchers explained the study and invited questions from children. Prospective participants were given a parent/child consent form to take home on the first day of, or prior to, the camp. The information pamphlet invited parents to contact the researchers directly if they had any questions or concerns. Incentives to participate were not offered. Camp staff regularly reminded children to return their signed consent forms to a centrally located box if they were interested in participating. Once parental consent was achieved, signed consent was also sought from children, to indicate their assent to participate. Eleven children aged 10–13 years (seven females and four males) indicated their interest in participating.

### 2.2. Participants

Participants included seven girls and four boys aged 10–13 years (One child aged 10 years, five aged 11 years, three aged 12 years, and two aged 13 years). Across the group, children described a range of diverse family structures and living arrangements. Four children lived permanently with both of their biological parents and siblings (i.e., nuclear family structure) and one participant lived with her grandparents and some of her siblings. Three households were blended (i.e., one biological parent and a stepparent) and three children lived in single-parent households. Over one quarter of households had other cohabiting adults present for short or longer periods of time, for example cousins, grandparents, aunts, uncles, or friends (n = 4). In most cases, cohabiting household members assisted by contributing financially (e.g., paying rent). Almost all households (n = 10) had two or more children. However, in some instances (n = 4), children had other older siblings who were living elsewhere, for various reasons.

### 2.3. Data Collection

Eleven one-to-one semi structured interviews were conducted by SV and SB on camp grounds between July-December 2018. Each interviewer undertook separate interviews with different children whilst at camp, then actively debriefed with one another at the end of each day. Interviews were undertaken in a private space such as an empty room or library and lasted between 20 and 40 min. The authors developed a semi-structured interview guide of 16 questions incorporating open-ended warm-up questions, food-related questions pertaining to shopping, cooking, and eating at home, hunger awareness, and questions on how hunger could be addressed. The semi-structured interview guide was developed via a three-stage process. Firstly, the researchers reviewed the qualitative literature on children and their experiences of food insecurity and explored the types of questions/areas covered in interviews. Secondly, drawing on the experience of our team, both in terms of qualitative interviewing with vulnerable populations and child-centred research, we further adapted the instrument to include interactional opportunities such as drawings and visual prompts, various probes and plain English phrases. Thirdly, the interview guide was piloted with two children aged 10–13 years who were not camp attendees. Minor modifications to the questions were made to ensure child-friendly language to maximise understanding before recruitment commenced. After the first four interviews, researcher feedback informed the development of new probes and refined ordering of the interview schedule. Children were encouraged to elaborate on their answers through numerous probes and close attention was paid to non-verbal cues.

**Figure 1:**
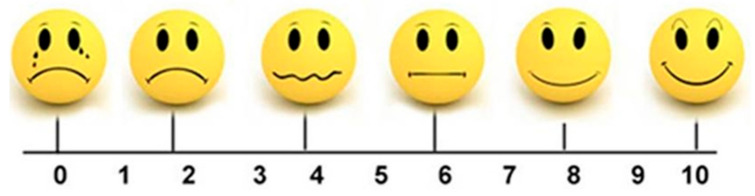
Emoji scale used in interviews.

Each child was provided with drawing materials and invited to doodle or draw at during the interview (e.g., to depict their favourite meal early in the conversation). The interviewer did the same in her own notebook and shared drawings with the child, to build rapport and ease children into the interview. In some cases, the child did not wish to draw, and the interview proceeded accordingly. In addition to drawings, the interviewers used a Likert scale containing emojis, small digital images used to convey an idea or feeling. The emoji scale depicted a range of expressions and emotions, including sadness, uncertainty, and joy (Figure 1). This technique supported children to engage in the research by enabling them to point to certain emojis to express their feelings pertaining to sensitive scenarios and circumstances (e.g., “What does being hungry feel like/How does this make you feel?”) [42]. Interviewers constantly sought clarification to delve more deeply into children’s meanings [41]. Children were also invited to stand up and move around halfway through each interview, to promote engagement. Recruitment ceased after the initial sample of 11 participants had been interviewed as, at this stage, theoretical saturation was achieved [43], i.e., no new ideas were emerging from the data set.

Interviews were digitally recorded and transcribed verbatim. Both interviewers also kept detailed field notes to capture the non-verbal aspects of interviews, as well as their own perceptions. These notes importantly served as an additional data source that enhanced the analytic process [41]. Given the interpretivist nature of this study, the positioning of the researchers as co-constructors of meaning was integral to the interpretation of data. Reflexivity was promoted and carried out in numerous ways, including the construction of field notes, as described above, and regular debriefing during data collection and analysis. These critical, sustained discussions enabled the researchers to support one another as sounding boards and ‘devil’s advocates’ [41] (p. 254) and allowed them to explore their own presence in the research. Given the sensitive nature of the research [44], debriefing also supported the researchers’ own emotional wellbeing.

### 2.4. Analysis

All data were imported into NVivo 12 (QSR International) to enable data management and coding. The children’s drawings were analyzed as an integral part of the data alongside transcripts. Firstly, the transcripts, drawings, and associated field notes were reviewed closely until the researchers were intimately familiar with the data. As new interviews were being conducted, earlier interview transcripts were coded and compared. SV and SB initially conducted line by line deductive coding of the transcripts, then these codes were grouped into key categories. Secondly, an inductive approach to coding was taken, with new themes emerging from and being grounded in the data [45]. Primary responsibility for the second round of coding was undertaken by SB, an experienced qualitative researcher. A process of inter-coder agreement was used for reaching maximum agreement on the codes and emergent themes, rather than inter-coder reliability as per Miles & Huberman [46]. The process of quantifying the degree of agreement was determined unnecessary as we were primarily interested in comparing the overall interpretation of text segments, rather than counting individual codes that were handled differently, or the number of variations in coded text units between members of the research team. During stage two, a subsample of transcripts was independently coded by other team members (SV, CP, and JS). Where our codes were divergent, we had a discussion and came to a consensus regarding re-defining a code and collapsing codes. The codes were then clustered into themes which were reviewed, discussed, and revised by the research team. Further details pertaining to nodes, codes, and examples of quotes can be found in the Supplementary Table S1.

## 3. Results

**Figure 2:**
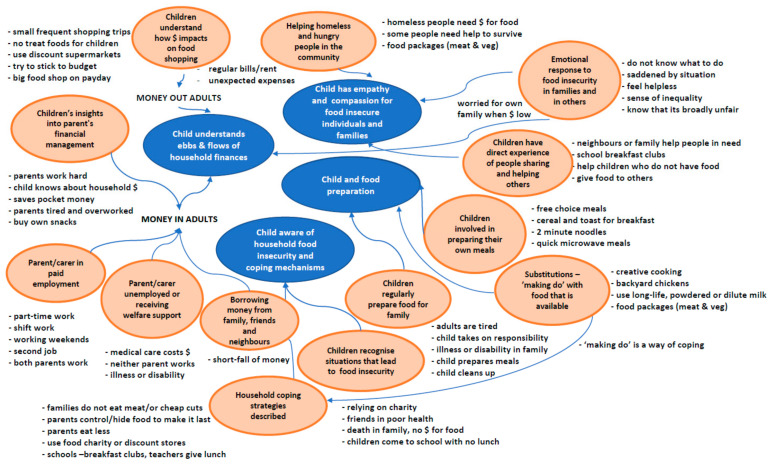
Thematic map of children’s perceptions, understandings, and experiences of household food insecurity. The map illustrates major themes (blue), sub-themes (orange), key ideas, and their connections (arrows).

Four main themes describing children‘s perspectives on food insecurity emerged from the analysis (Figure 2). Children had; (i) financial understanding, (ii) awareness of food insecurity and coping mechanisms, (iii) sharing, empathy, and compassion for food insecure families, and described (iv) the nature of food preparation. For confidentiality, pseudonyms are applied as necessary for the children’s quotes.

### 3.1. Theme One: Financial Understanding

Most children demonstrated a sophisticated understanding of their parents’ finances, including income and expenditure, and the temporal nature of how money ebbs and flows through the household. Children’s discussion of income largely centered around the employment status of caregivers. Employment in families was highly variable and work was sometimes precarious. Circumstances included both parents working full-time; part-time workers; workers with several jobs; unemployed but actively seeking work; or those reliant entirely on welfare payments or worker’s compensation. In many cases unemployment was long term. For example, when Samantha was asked how long it had been since her mum, stepdad, or her stepdad’s brother were able to get a job, she responded, “It’s been about five or six years”. Another girl similarly explained that her dad had been out of work for six years, after enduring a workplace injury.

Further to employment status of caregivers, children’s knowledge extended to financial tensions at home. In some instances, a shortfall of money combined with the arrival of bills and unexpected expenses, such as healthcare, or cost-laden holiday periods were noted as stressful. In their discussions, children demonstrated an acute awareness of how money impacts on the availability of resources for the household and their needs and desires.

“The bills and all that is why we don’t have enough money because we have to pay them, and we only end up with around AD$200 at the moment.” (Cathy)

Most children described how their household operated on a food budget when shopping for groceries. Shopping trips were very much shaped and defined by the amount of money available and the timing of payday cycles. Samantha described one shopping trip where her mother ran out of money at the checkout.

“So, Mum got everything. She even got the chicken. She put it on the counter but then she heard that it was $25 but she only had $22 on her so she had to put one [item] back, and the pies were only $3, so she got that instead of that.” (Samantha)

When shopping with parents, some children refrained from requesting certain foods because they knew they were costly, or because they were aware there was insufficient money to buy all the ingredients for a favourite meal. Implicit in the children’s discourse around financial awareness was a sense of acceptance, or that there was no choice in the situation—it was just how things worked. As Cathy explained,

“Because we don’t have all the ingredients [for spaghetti bolognaise] most of the time because my Dad doesn’t get a lot of money per week, so he’d only get, like, the stuff [food] that really matters. Like, school food and stuff for dinner, then rice and dog food for the dog and cat food for the cat...” (Cathy)

When questioned about how she felt about not having regular access to favourite foods, Cathy described her acceptance of having fewer food choices available. She said she sometimes had to refrain from eating food from the cupboard when she felt hungry or was limited in what she had available to eat. In a calm tone she described,

“I feel okay because I know that I’m going to have something to eat later and I’m not going to starve to death…. you can’t always get what you really want and even if it’s as little as food, even if you have, like, a sandwich a day and you really want something else, that’s okay because your parents might surprise you one day…” (Cathy)

Another child living in a sole parent family with four other siblings, described his mother’s urgency to withdraw her entire welfare benefit from her bank account before various automatic bill deductions were made. If she managed to get to the automatic teller machine early on the day the money was deposited, she could withdraw all the funds and have sufficient money for groceries. If not, they would only be able to afford food from the low-cost discount Dollar Shop.

Alongside children’s demonstration of economic awareness there were other examples suggestive of economic anxiety. Children’s understanding often extended to insight into the level of sacrifices that parents made to keep the household financially viable by navigating low incomes, rising costs of living and unanticipated expenses. Some participants noted their parents were working long hours, and sometimes in a second job or on the weekends, to earn enough money for food and other expenses, which seemed to cause stress and fatigue for parents. Children expressed understanding and a sense of appreciation for their parents’ work.

“Sometimes I see her [Mum] rushed because of little ones [younger siblings] and she’s got to do that…. Dad doesn’t cook, because he works and plus, he’s tired because he starts like 3 a.m. shifts and stuff, like 2 a.m.…” (Bruce)

In some, but not all interviews, there was also a sense of worry and sadness as children recounted their parents’ efforts to manage, and the impact this had on their families. For example,

“.we got a letter and it said, and our gas bill was so much, it was like AD$1000, because we use so much gas in our house. Our house is really cold! We don’t have much stuff on our walls [Insulation]. Mum only had AD$300, and Dad had AD$800, so the money that we had there, we had to pay, that’s how we paid it and we only had $100 left… My dad had to get another job and so that’s how we get our money now.” (Ingrid)

“I’m scared that we’re not going to have enough money to buy more food. Because my mum, she pays everything on what she wants, usually, but then she pays us something [pocket money], but then there’s nothing for groceries, so that means that we only had to stick with the pasta for a few days…. I feel like scared and we’re going to lose our house…. Mum only pays [the bills], like AD$250, every single month, and sometimes she doesn’t have any money because she paid the bills, and then we don’t have any groceries, and our bills keep going higher and higher and higher, so we don’t get any groceries that often... I’m scared if I tell anyone it will be a big deal.” (Samantha)

### 3.2. Theme Two: Awareness of Food Insecurity and Coping Mechanisms

Children described episodic food insecurity in various forms, for example, in the community, and amongst known individuals, including extended family members, parents’ friends, or students who went to school ‘with nothing to eat’. In discussing food insecurity within the broader community, and some of the drivers that influence food choice, children referenced broader social issues such as homelessness, people having different amounts of money (social inequality) and unemployment. In response to the question “Are there any other things that families can do if they don’t have enough money for food?”, some children responded that they could get a job, ask people for money, stop smoking, or eat smaller quantities. Throughout the course of the interviews, it became evident that episodes of food insecurity also occurred in some of the children’s own households, as they shared their own experiences of restricted food choice and availability.

Researcher: “Sometimes in Australia there are families that don’t have enough money for food.”

Participant: “Yeah, that’s kind of that what happens to us sometimes, like right now at home we only have like this much milk right now [gestures], and Weetbix [breakfast cereal] and I don’t know how much bread we have. We actually have no food right now…” (Theresa)

Sometimes food choices were limited due to insufficient money because of limited employment hours or recent payment of bills or medical expenses. For example, Tracey described the impact of a recent visit to the dentist.

“She’ll [Mother] be like what do you want for dinner hon, and then sometimes Dad doesn’t realise that Mum hasn’t got much work because she must’ve taken a day off to take my sister to the dentist or something. She’d be like what do you want kids and she’d be like oh, we can’t tonight, I didn’t get a good pay because remember when I took [sister] to the dentist. And also, she had to pay for the dentist as well.” (Tracey)

Based on the children’s narratives, household composition and the variable numbers of people within a household at any given point also influenced food availability. For example, Kelly described her father going without food due to limited resources when additional people were visiting the household, explaining:

“Dad rarely gets any [dinner], so, if there’s people at home …there’s nine people in my house right now. My Nan’s friend that’s staying with us for a couple of days because, because he drove her back to here because she, she was on holiday, so we got tacos. And then my brother’s friend Ben. He stays here for a while. Then my brother. Lauren, my sister which is Lauren. My Mum.” (Kelly)

While fluctuating financial resources placed strain on families and sometimes impacted on food availability, children described various coping mechanisms adopted. For example, when money was available, some families would stockpile cheap food items on ‘special’ or use tax return refunds to buy extra pantry staples. By contrast, when food was scarce due to financial restraints, children described other strategies that adults used to maximise the use of available resources. These included buying out of date food; rationing food; borrowing money from friends and neighbours; creative cooking with cheaper pantry staples or available foods (e.g., chicken eggs); shopping at discount stores; bringing home leftover foods from work (e.g., aged care home), or accessing food relief from charities. The quotes below were typical of coping responses described by the children:

“My nanna Di. She eats less…. Yeah. And my nanna Valerie,], too…. Yeah, my nannas, what they do is my nanna, whenever she gets her money, she buys a lot of food, and she puts it in little plastic bags… whenever she gets her money, she buys a lot of food, and she puts it in little plastic bags. Then one night she’ll have what she put in there, and then the next night she’ll have another one, and then she does that until she gets paid again.” (Ingrid)

“But when she [Mum] gets the big money in July, or June [tax refund], she does the big shopping, like, a big big shopping, because we eat all the food.” (Bruce)

“…It’s [the food] gone in three days because we eat it. Yeah. We eat it all…. Then we be imaginative. We put things on the barbecue and do a lot of things. We have eggs because we’ve got chickens.” (Kelly)

“They [the church] give us—they have all like these donuts and like fruit, muffins and stuff in there. And they would give you bread, milk and stuff... They give you something to eat at least.” (Theresa)

In certain cases, parents tried to ration the food that was available, or ate less so that children could eat more.

“[Dad’s’] a really nice person because he’ll dish up everyone else and then if there’s any left over he’ll have it. And if there isn’t enough then he’ll, he’ll either miss out because he’s not hungry or he’ll, make some toast if there’s any bread. [I feel] not very good because while I’m eating some really nice stuff my Dad’s just having toast every night for dinner.” (Kelly)

Some children also had to seek their parents’ permission each time they wanted to eat food such as snacks from the pantry. For one child, taking family food was considered stealing, as they said their parents tried to control access so that the food would last until the next shopping day..

“No, ‘cause I’m not allowed to [access food freely if hungry], I have to ask Mum ‘cause if I get up and get some food mum reckons it’s called stealing food.” (David)

“I ask my dad if it’s okay to grab something to eat. He usually says yes. Then, like, when he’s angry when [sibling’s name] ate all the stuff in the cupboard he says no because we’re just going to eat it all.” (Cathy)

One child described hoarding food in his bedroom to avoid his siblings raiding his food supplies from the cupboard.

“Kevin tells me he has a bar fridge in his bedroom where he keeps the food he’s bought e.g., bags of apples, soft drink etc. He says he spends a lot of time in his room because his siblings will raid his fridge and steal/eat his food. He tells me he’s saving up for a safe ($40) so that he can lock his food away safely and be able to leave his bedroom” [Field notes, SB]

### 3.3. Theme Three: Sharing, Empathy and Compassion for Food Insecure Families

Children were acutely aware of the lack of equity in the world and described a sense of compassion towards others who needed support due to not having enough money for food. There was a general willingness to help others, and this was articulated in the provision of food, money, shelter, and employment opportunities. Some children demonstrated a sophisticated ability to show compassion to others experiencing food insecurity such as homeless people. Several children described how they shared their own lunches with friends who had come to school without enough food.

“They [children who come to school with insufficient food] might feel upset because if they —like, when they go to school, and they see everyone else getting their lunchboxes out for recess and they don’t have anything for their own they might feel upset and jealous maybe? Yeah, I’ve seen a couple of kids at my school have nothing to eat so I usually give them, like, half of my sandwich to make them feel better. They feel really happy afterwards because they get something to eat.” (Cathy)

Established informal mechanisms of support and reciprocity between neighbours and friends, such as helping each other out during times of hardship, or lending money, were also described by children. A sense of caring and helping others became evident throughout the interviews:

“Yes. That’s sort of we have like our neighbour next door...sometimes they won’t have enough money and ask, and they would ask us if we could—they’d ask us politely if we could get some food, if we can get some things for us. But sometimes we ask them politely as well, ‘cause sometimes we get too low, or mum forgets to go to get the things [food].” (Colin)

Children also described being emotionally impacted by others who had less food or were disadvantaged and indicated sadness, uncertainty or helplessness using the visual emoji’s during the interview. For example, Bruce proclaimed, “that makes me upset, that they got on the street struggling”. Other children commented:

“I have noticed a boy who used to go to our school, he lives just over there, he used to ask me every day for food. I was like, why do you need food, and he’s like, because I’m really hungry, and started crying. I felt really upset, I started crying so I gave him my recess [snack].” (Tracey)

“Well, maybe from my friend Jack, from school. I haven’t been to his house, but whenever school happens all he has is his sandwich, so, for like recess, he has to ask the teachers all the time [ for food for lunch] …. I don’t really know [why it’s happening]. He doesn’t really say anything [about his situation] …. It makes me feel, like, not sure… [points to number 6 on emoji scale—‘uncertainty’] because I don’t know what to do…” (Kevin)

Children’s deep compassion was also reflected in discussion of the possible solutions to food insecurity, including the roles and responsibilities of government to help others less fortunate, charitable responses and philanthropy. While a few children provided whimsical responses about providing people with “money trees”, or packages of food appearing at people‘s doors, Kelly eloquently and sincerely expressed the need for more equity in the world:

“I want everyone in the world to be the, well not the same as in like the personalities and things but be treated the same and have the same amount of everything. So, if there wasn’t enough money people would actually share the money. They wouldn’t just take it all for themselves. So, everybody would get equal money and stuff.” (Kelly)

Following this interview, the researcher reflected, “[she] was acutely aware of food hardship and explained her wish for things to be fairer… I felt immense sadness in response to [her] story and wished I could change things for this girl” (SV, field notes). In expressing their ideal worlds, Cathy and Bruce offered solutions involving increased government spending, access to quality housing, and employment training.

“I would give them [families] more money and more food to live off of and then I’d donate some more money to charity for those in need.” (Cathy)

“If I was the government, I think to help all people who need the money or food. You see homeless people on the street, to help them… And I think the government should help, with the charities…Maybe get, start off with a small amount of job and then try and like get a different job that pays more or less if you get a raise…I think the government should hear this.” (Bruce)

### 3.4. Theme Four: The Nature of Food Preparation

Children provided detailed insight into cooking practices and the types of food consumed in their households. Many were involved in food preparation and cooking tasks, particularly at breakfast. Sometimes when groceries were running low, children were resourceful, preparing alternatives for themselves:

“Well sometimes if we’ve run out of milk, we’ll have toast [for breakfast], but if I have milk…if there’s like a full carton of milk, you know those bottles, not like those cardboard ones [long life milk]… I would make, I mainly eat Weetbix [cereal] … And I have Weetbix with hot water and milk” *(Colin)*

Some children were tasked with preparing dinner, particularly when parents were tired after a day at work. These meals often consisted of quick, easy, and simple to prepare foods that were cheap (e.g., chips and nuggets) and high in carbohydrates (e.g., macaroni and cheese). David described he and his brother having ‘free choice’ dinners around twice per week, which often comprised a cup of soup, noodles, microwave chicken rice, or canned spaghetti.

“Yeah, when mum and dad are tired, they make us pick our own then they say we can cook it.” (David)

Cathy similarly described preparation of a common meal she would make for herself and her father, using the microwave:

“...I may not necessarily have a lot of money to order pizza every single night, so I need to learn how to cook... So, basically, I put two packets of noodles in so I can split them in half and give some to dad as well, so he doesn’t go hungry. And then I put them in the microwave or just in the boiling hot water and then, yeah, it’s done.” (Cathy)

In some instances, children or their siblings were “over responsible” and offered additional support to parents who were fatigued or unwell by taking a more active role in food provision and other responsibilities. Theresa’s quote below illustrates her desire to “look after… everyone” and she referred to her mother who had a chronic illness, as well as other family members who were sick or injured at this time.

“Jackie [sibling] had a broken leg... and I had to get her food and stuff, had to get mum’s food, ‘cause I had to look after mum. Because [stepdad’s brother] was sick I had to look after [him], everyone. Do the dishes, clean everything. And then I’ll come to school and then be tired, and everyone’s yelling at me…” (Theresa)

## 4. Discussion

The study describes how children living with socioeconomic disadvantage articulate the characteristics and their experiences of food insecurity. It is important to expose how these experiences of poverty negatively impact children and blur the boundaries between childhood and adulthood [47]. The findings show children are active agents in shaping and making sense of their lives, even in times of significant disadvantage. Firinci Orman’s concept of adultization breaks down the binary of either negative or positive framings of modern children’s experiences. Rather, as our results reflect, food insecurity presents children with both ‘dangers and opportunities’ [47] (p. 111).

Overwhelmingly, results from this are consistent with existing research. Connell et al.’s study of children’s perceptions of food insecurity found children’s understandings could be categorised as relating to quantity or quality of food, and emotional and social wellbeing [25]. The children in our study similarly described restricted amount of food available at home and how the quantity and quality of food available could operate in waves. For example, food was in surplus shortly after pay day and strategies were put in place to maintain availability through to the next pay.

As reflected in Harvey’s study with 5–11-year-old children in the UK [24], our participants described household strategies to ensure consistent food supply, suggesting that parents were sometimes unable to afford a nutritious diet and hence sought out cheap, filling foods to stretch their money further. Children described consuming more cheap food like pasta, and less fruits, vegetables, and dairy every day. Researchers hypothesise that consuming low-cost energy-dense foods, in the absence of sufficient food, can produce a tendency to over-eat when food does finally become available [15]. Compromised dietary quality is a consequence of food insecurity [15] and the children’s narratives support this. Many busy, time poor or exhausted parents tasked some children with preparing easy meals. This finding points to the elements of time and employment schedules, which are often overlooked in policy discussions of family food insecurity, given the emphasis on economic insecurity. As Gallegos explains, “time and money are both needed to manage a household where money is lacking” [48]. These findings support the complex interaction between income, employment, and food.

Harvey [24] and Connell et al. [25] also found parents were actively shielding their children from hunger and other effects of food insecurity. Some children in our study described how their parents prioritised serving the evening meal to the children first and only eating themselves if sufficient food remained. The protective action of parents illustrates what Firinci Orman [47] describes as them operating in a risk society, constantly seeking to shield children from the realities of disadvantage and global/local threats. Yet at the same time, our results also provide an empirical example of the “democratisation of the family” [47] (p. 109), where children are active citizens involved in shaping family and community life. Our results show how children are themselves actively engaged in protecting their peers and family, including adult relatives, from hunger. For example, Cathy talked about cooking two packets of noodles to make sure her father did not go hungry. This idea echoes the work of Fram et al. [14] who found that children took on significant levels of responsibility for managing food resources, and the finding of Gunson et al. [28] that managing hunger is not just embodied but also carries relational and political significance.

Children are frequently involved in developing coping strategies to manage food supply and will often learn these through observation of the adults around them [25]. Our findings show how this occurs at times, but also how children develop their own strategies and enact agency within their families. In Cathy and Theresa’s stories, we observed that children can take on caring roles themselves that involve significant responsibility for the adults and children around them, particularly when their own parents are unwell. This speaks to literature on the life course, for example Swan et al. [49], that demonstrates how key episodes of adversity can lead to the development of coping strategies that improve social capacity for food-related wellbeing in adulthood. Indeed, the way in which children engage and develop innovative coping strategies could be interpreted as empowering, for example where children are able to have insight into a complex problem and develop critical skills to remain resilient in the face of vulnerability. An awareness of family finances and implications for food supply might support the development of financial literacy, particularly where children observe the practices of parents who are economically resourceful. Such skills could reasonably support children in their adult lives to maximise the use of available resources, should food insecurity arise. However, the social and emotional toll on the participants in our study cannot be overlooked, particularly when we consider the worry and concern that some children expressed. This supports findings by Leung et al. [27] that the necessity of developing coping strategies for children experiencing food insecurity can contribute to anger, frustration, and psychological distress. From this perspective, childhood food insecurity could be conceptualised as a distinct form of adultization, whereby necessity to navigate ‘adult’ knowledge and mature forms of stress might impact on children’s opportunities for learning, creativity, and recreation, thereby widening inequalities.

Indeed, the impact of food insecurity on the emotional and social wellbeing of children and their peers in our study was palpable. Our findings are consistent with Fram et al. [14] and Connell et al. [25] as our participants were emotionally and physically aware of their own and others’ experiences of food insecurity. They described feeling sadness as a result of their awareness of the hunger experienced by those around them, for example, their peers. This level of awareness also related to financial matters and the complex ways in which money had to be managed to try to ensure that food could be accessed throughout income cycles. As in Ghattas et al.’s study with Lebanese children [26] the Australian children in our study perceived lack of money as a primary driver of food insecurity, and expressed empathy related to food insecurity. In our work, the potential harm of having such heightened awareness came through in Samantha’s description of her fear of losing her home, as well as Ingrid’s account of the cold she felt because of not being able to afford heating. However, unlike the participants in studies by Connell et al. [25] and Lueng et al. [27], children in our research did not describe any sense of shame associated with poverty or food insecurity. By contrast, some participants demonstrated clearly formed perspectives on the issue of poverty and the relationship between money and food security. In expressing their views on economic and social inequalities, some participants highlighted the importance of governments and/or communities sharing and providing adequate resources and opportunities to support each other. They also acknowledged reciprocal community relationships that enabled them and their families to support others and be supported when necessary, which echoes Leddy et al.’s [50] recent analysis of social capital and food insecurity. Overall, our participants reaffirmed our and other’s arguments that children can make meaningful contributions to research, and indeed, their voices can shape practice [51] and address marginalisation [52]. Neve et al. [39] explain that drawing on lived experience evidence to shape food systems can mean the difference between policy success and failure. In this case, the participants’ empathy, strategies, and solutions demonstrate that children should be involved as experts in co-design of public health interventions relating to food. As is starting to occur in other areas of policy, children’s lived experience needs to be recognised as expertise and put at the centre of system redesign [53].

Previous studies have called for research to focus on inclusion of children as participants in research that seeks to unpack the complex problem that is food insecurity [14,23]. Our study has responded directly to such calls and confirms that children are important voices in providing profound, fine-grain evidence of the impact of socioeconomic disadvantage on food access. The children in this study understood food insecurity in relational ways and their experiences demonstrate the ways in which adultization of childhood presents both dangers and opportunities for children as family members and, more broadly, as citizens [47]. The accounts showed that, whether through first or second-hand experience, children described food insecurity through their relationships with other people, through concern and empathy for peers, fear of siblings taking their food, the ebb and flow of house guests coming and going, or care and concern for a hungry father. These accounts underline that food or lack of food is a relational issue and suggest that attempts to address this issue must acknowledge children’s awareness of how food insecurity impacts not just themselves but those around them. Our findings are also significant in that they demonstrate the importance of considering how age and intergenerational relations are a significant social variable [33] that shape the ways in which food insecurity is experienced. In this study, we saw evidence that the modern nuclear family is no longer “typical” [47] (p. 109), which influences the ways in which children and families navigate food insecurity. Food insecurity is clearly a ‘real’ and complex issue for Australian children and our work lays the foundation for further child-centred studies in diverse contexts, to generate a more nuanced understanding of this phenomenon.

To our knowledge, this is the first Australian study that specifically investigates the experience of food insecurity from children’s own perspectives. The child-centered nature of this study, and the incorporation of visual tools, should be viewed as clear strengths, as they supported the generation of rich, nuanced data. Considering the sensitive nature of this research, and the well-documented shame associated with food insecurity, it is possible that some participants withheld certain views to gain the acceptance of the researchers [54]. While this is a potential limitation, we actively implemented several strategies that sought to build rapport, minimise power imbalance, and create a warm, supportive research environment to reduce the likelihood of this occurring.

## 5. Conclusions

Australian children are aware of, experience, and are clearly impacted by food insecurity. In this study, children expressed ideas about the experience of economic disadvantage and a clear awareness of, and empathy for, the realities that families face due to the cost of living. Listening to children’s stories allows for a deeper understanding of what food insecurity means for children in different contexts. This study sheds light on the untenable and uncertain circumstances in which some children find themselves. Our participants described feelings of sadness and concern associated with food insecurity and described numerous coping skills adopted by those around them. Future research should seek to extend child-centred work. Mixed methods studies will play an important role in quantifying food insecurity and allowing for more generalisable results, while consolidating the understanding of children’s lived experiences and coping strategies adopted across diverse contexts. Importantly, a comprehensive understanding of childhood food insecurity can provide key stakeholders with a knowledge base from which to anchor program and policy responses that are in the best interests of children, by taking their needs and experiences into account.

## Supplementary Materials

The following are available online at https://www.mdpi.com/article/10.3390/ijerph18084039/s1, Table S1: Data Codebook extract from one theme—Examples of nodes, codes, and supporting quotes.
