# Supplementary material for: How Do Disadvantaged Children Perceive, Understand and Experience Household Food Insecurity?
Source: Int J Environ Res Public Health. 2021 Apr 12;18(8):4039. doi: 10.3390/ijerph18084039 (PMC8070068; doi:10.3390/ijerph18084039)
Supplement: Supplementary file 1 [file ijerph-18-04039-s001.pdf]

International Journal of
Environmental Research and Public Health

MDPI

Table S1. Data Codebook extract from one theme – Examples of nodes, codes, and supporting quotes.

Theme	Node	Code	Examples of Quotes
**Awareness of food insecurity and coping mechanisms**	Coping strategy	Borrowing money/food from other family members	*"...our neighbour next door …sometimes they won't have enough money and... they'd ask us politely if we could get some food…. But sometimes we ask them politely as well, 'cause sometimes we get too low [in food…"*
			"I: So what do you do in those circumstances, apart from eat lots of pasta? P: Well, we usually go to XXXX's [Older sibling in full time employment] for dinner or XXXX gives us some money."
			"We get my step mum to pay for the pizza so then there's enough for everyone because, like, my dad can't afford it most times when there's a lot of people".
		Creative cooking, i.e. using pantry items or what is available	*"He gets a big shop, like, he goes and gets everything that he needs, puts them in the cupboard. It's gone in three days because we eat it... Then we be imaginative. We put things on the barbecue and do a lot of things. We have eggs because we've got chickens."*
	Children aware of parent coping strategy	Parents controlling child's food to make food last	*"I: What do you do if you're ever hungry at home? P: I ask my dad if it's okay to grab something to eat…He usually says yes. Then, like, when he's angry like when XXXX [Sibling] ate all the stuff in the cupboard he says no because we're just going to eat it all."*
			"I: …you told me you once felt hungry in the night, what would you do then? Would you get up and get some food? [Participant shakes his head]. I: Why not? P: No, 'cause I'm not allowed to, I have to ask mum 'cause if I get up and get some food mum reckons it's called stealing food."

	Children notice use of food charity, discount supermarket or dollar shop instead of major supermarket chain for cheap food or free food when money is low	*"It happens normally a lot. So we go to a church and sometimes the church comes out of nowhere and they invite us: They give us – they have all like this donuts and like fruit, muffins and stuff in there. And they would give you bread, milk and stuff".*
		"[Mum] goes to Rexi's… It's a little small shop but it's okay, and it's not as dear there. So you can get like – you can get a massive watermelon for like 99 cents."
	Child awareness and anxiety about food, e.g. parent deliberately eating less and running out of money for food	*"….he'll dish up everyone else and then if there's any left over he'll have it...And if there isn't enough then he'll, he'll either miss out because he's not hungry or he'll, he'll make some toast if there's any bread…[I feel] Not very good because while I'm eating some really nice stuff my Dad's just having toast every night for dinner."*
		"Because my mum, she pays everything on what she wants, usually, but then she pays us something [pocket money], but then there's nothing for groceries, so that means that we only had to stick with the pasta for a few days."
